# Innate Inspiration: Antifungal Peptides and Other Immunotherapeutics From the Host Immune Response

**DOI:** 10.3389/fimmu.2020.02177

**Published:** 2020-09-17

**Authors:** Derry K. Mercer, Deborah A. O'Neil

**Affiliations:** NovaBiotics Ltd., Aberdeen, United Kingdom

**Keywords:** antimicrobial peptide, host defence peptide, innate immunity, antifungal, immunotherapeutics

## Abstract

The purpose of this review is to describe antifungal therapeutic candidates in preclinical and clinical development derived from, or directly influenced by, the immune system, with a specific focus on antimicrobial peptides (AMP). Although the focus of this review is AMP with direct antimicrobial effects on fungi, we will also discuss compounds with direct antifungal activity, including monoclonal antibodies (mAb), as well as immunomodulatory molecules that can enhance the immune response to fungal infection, including immunomodulatory AMP, vaccines, checkpoint inhibitors, interferon and colony stimulating factors as well as immune cell therapies. The focus of this manuscript will be a non-exhaustive review of antifungal compounds in preclinical and clinical development that are based on the principles of immunology and the authors acknowledge the incredible amount of *in vitro* and *in vivo* work that has been conducted to develop such therapeutic candidates.

## Introduction

Medical and technological advances, improvements in hygiene and availability of vaccines to important life-threatening diseases means that since 1900 global average life expectancy has more than doubled and is now more than 70 years (1). Despite this, the prevalence of both life-threatening and superficial fungal infections has increased and has largely coincided with progress in the treatment of other diseases (2). Systemic fungal infections are significant causes of morbidity and mortality, responsible for the deaths of more than 1.6 million people per annum (3); comparable to tuberculosis and more than 3-fold higher than malaria. All fungal infections have risen in prevalence over recent decades, including allergic bronchopulmonary aspergillosis (ABPA) and superficial fungal infections, with the increased use of immunosuppressive medications for cancer and transplantation and patients with HIV/AIDS and other immunodeficiences (including genetic disorders), as well as indiscriminate antibiotic use, parenteral nutrition and permanent indwelling catheters. Climate change, pollution and environmental disruption are also considered likely to contribute to the increased incidence of fungal infection and fungal antigenicity (4–7). Defects in innate immune responses, including neutropenia, alveolar macrophage dysfunction, and mutations in STAT3 (resulting in autosomal dominant hyper IgE syndrome) and impaired NAPDH oxidase activity facilitate the development of pulmonary, and in some cases invasive, aspergillosis (8), whereas mutations in the gene for CARD9 (signaling adaptor protein for the C-type lectin receptor) results in increased susceptibility to many types of fungal infection, including dermatophytosis (9, 10). The reasons for the increased incidence of fungal infections over recent decades are beyond the scope of this manuscript and readers are directed to several excellent reviews on the subject (3, 4, 7, 11–15). Fungi are ubiquitous throughout nature and we are constantly exposed to these microbes from the environment via inhalation, ingestion or on epithelial surfaces including the skin and mucosae (16–21). Most fungi are not pathogenic to humans, and most of those that are do not cause life-threatening infections in immunocompetent individuals and such infections are relatively rare. Of the fungi that are able to colonise the human body, many co-exist (commensalism) without causing infection under normal circumstances, e. g. *Candida* spp. (22, 23). *Candida* spp., (~750,000 cases of invasive candidiasis/year) *Cryptococcus* spp. (~225,000 cases per annum in AIDS patients/year) and *Aspergillus* spp. (~3.75 million cases of chronic pulmonary or invasive aspergillosis/year) are responsible for a significant number of life-threatening fungal infections, whereas other fungi are responsible for substantial levels of systemic infection, including *Pneumocystis* spp. (~500,000 cases/year), *Histoplasma* spp. (~500,000 cases/year), *Coccidioides* spp. (~25,000 cases/year) and mucorales (>10,000 cases/year) (4, 11). Fungi cause superficial infections of the skin, hair, nails and mucosal membranes, including dermatophytes, *Candida* spp. and *Malassezia* spp. that are normally readily treatable. There are at least 1 billion cases of superficial fungal infection each year and this is both under-reported and increasing in incidence (3, 24). Dermatophytes are the main cause of superficial fungal infections and each year 20–25% of humans and animals suffer dermatophyte infections (25). Fungal exposure is also thought to contribute to allergies and worsening of asthma symptoms (e.g., ABPA), affecting millions of individuals worldwide (8, 26, 27). Difficulties in diagnosis, the limited antifungal armamentarium, the lack of any fungal vaccines and our limited understanding of the immune response to fungal infection all contribute to this disappointingly high level of morbidity and mortality (Table 1).

**Table 1:** Human fungal infections, incidence and treatment options [adapted from (3)].

**Infection**	**Fungus**	**Infection type**	**Infection site**	**Incidence (cases per annum/global burden)**	**Therapeutic options**	**Reference/s**
ABPA^1^	*Aspergillus* spp.	Allergic	Lung	~5 M (GB^2^)	Glucocorticoids ± itraconazole	(28)
Pulmonary aspergillosis^3^		Severe	Lung	~3 M (GB)	Voriconazole, itraconazole	(29)
Invasive aspergillosis		Severe	Disseminated	>300 K	Voriconazole	(30)
Oropharyngeal candidiasis	*Candida* spp.	Mucosal	Mouth	~3.3 M	Oral nystatin, miconazole or clotrimazole^4^	(31)
Vulvovaginal candidiasis		Mucosal	Genitourinary tract	~134 M (GB)	Topical antifungal, fluconazole	
Invasive candidiasis		Severe	Disseminated	~750 K	Echinocandin, fluconazole	
Cryptococcosis	*Cryptococcus* spp.	Severe	Lung, CNS^5^, disseminated	~225 K	Fluconazole, amphotericin B + flucytosine	(32)
Tinea	Dermatophytes (e.g., *Trichophyton rubrum*)	Superficial	Skin, hair, nails	>1,000 M (GB)	Terbinafine, itraconazole	(33)
Severe dermatophytosis		Invasive	Disseminated	Very rare	Terbinafine, itraconazole, posaconazole	(34)
Mucormycosis	Mucorales (e.g., *Rhizopus oryzae*)	Severe	Rhinocerebral, lung, skin, disseminated	>10 K	Amphotericin B, posaconazole, isavuconazole	(35)
Chromoblastomycosis	Chaetothyriales (e.g., *Exophiala dermatitidis*)	Severe	Skin	>10 K (GB)	Itraconazole, terbinafine, posaconazole	(36)
Coccidioidomycosis	*Coccidioides* spp.	Severe	Lung, skin	~25 K (GB)	Fluconazole	(37)
Paracoccidioidomycosis	*Paracoccidioides* spp.	Severe	Lung	~4 K (GB)	Itraconazole, amphotericin B	(38)
Histoplasmosis	*Histoplasma* spp.	Severe	Lung	~600 K	Itraconazole	(39)
Sporotrichosis	*Sporothrix* spp.	Severe	Skin, lung, disseminated	>40 K	Itraconazole, amphotericin B	(40)
*Pneumocystis jirovecii* pneumonia	*Pneumocystis jirovecii*	Severe	Lung	~500 K	Trimethoprim/ sulfamethoxazole	(41)
Eumycetoma	Fungi (e.g., *Scedosporium* spp.)	Severe	Skin	~9 K (GB)	Itraconazole	(42)
Fungal Keratitis	Fungi (e.g., *Fusarium* spp.)	Superficial	Eye	~1 M (GB)	Voriconazole	(43)
Fungal rhinosinusitis	Fungal antigens	Allergic	Lung	~12 M (GB)	Corticosteroids	(44)
Talaromycosis	*Talaromyces marneffei*	Severe	Skin, lung, liver, disseminated	~8 K	Amphotericin B, itraconazole, voriconazole	(45)

1 *Allergic bronchopulmonary aspergillosis*.

2 *Global burden*.

3 *Includes aspergilloma*.

4 *For more severe cases oral or intravenous fluconazole can be administered*.

5 *Central nervous system*.

There is a limited armamentarium of antifungal drugs for the treatment of fungal infection and significantly, drug resistant fungal infections are emerging as important clinical challenges (46–51). Currently available antifungals fall into a limited number of classes; polyenes (e.g., amphotericin B and nystatin), azoles (e.g., fluconazole, itraconazole, voriconazole, isavuconazole, efinaconazole and posaconazole), echinocandins (e.g., caspofungin, anidulafungin and micafungin), allylamines (e.g., terbinafine and naftifine) and other lesser used or topical therapies including flucytosine, ciclopirox olamine, tavaborole, amorolfine, butenafine, griseofulvin, tolnaftate and natamycin. Most serious fungal infections are treated with drugs from only 3 classes; azoles, echinocandins and the polyene, amphotericin B (46, 52) and therefore, resistance to one class of antifungal limits treatment options to a significant degree (51). Whilst resistance rates are low compared to those, for example, of the bacterial ESKAPE pathogens, ~3% of *A. fumigatus* are resistant to more than one azole, whereas 1.0–1.5% of *Candida* spp. are resistant to echinocandins and rates of resistance are increasing (47, 48, 51). Analogous to antibiotic resistance, antifungal resistance may be caused by acquired resistance mechanisms as well as primary resistance (also referred to as inherent resistance). For example, azole antifungals inhibit the ergosterol biosynthesis pathway (an essential component of the fungal cell membrane) by targeting lanosterol 14-α-demethylase, encoded by Erg11 in yeasts and Cyp51A/Cyp51B in filamentous fungi. Resistance to azole antifungals can be as a result of over-expression of the target gene (*ERG11*), loss of function of other enzymes involved in ergosterol biosynthesis (e.g., Δ-5,6-desaturase enzyme Erg3), up-regulation of multidrug transporters (e.g., Cdr1, Cdr2 and Mdr1 in *Candida* spp.), genome plasticity causing chromosomal duplications (aneuploidy) and the inherent resistance of *C. auris* to fluconazole (48). The recent emergence of *C. auris*, a predominantly nosocomial pathogen first isolated from a patient in 2009, is associated with high rates of mortality and antifungal resistance. In the US ~90% of *C. auris* isolates are fluconazole resistant, 30% are amphotericin B resistant, although <5% of isolates are resistant to echinocandins. Additionally, multi-drug resistance of *C. auris* has commonly been reported, as has its ability to persist following disinfection of surfaces (49, 50, 53).

Clearly, new therapeutic options for the treatment of fungal infections are urgently needed (54). The global antifungal drug market was valued at US $11.92 Bn in 2018 and is expected to grow to US $13.87 Bn by 2026 (fiormarkets.com, 2020)^1^. Understanding the immune responses to fungal infection is essential for the rational design of more effective therapies and therefore improved patient outcomes in the future. Depending on the site and type of infection, the immune response can mount fungus-specific and/or site-specific antifungal responses. The development of antifungal drug candidates that replace or correct defective elements or dysregulation in appropriate immune responses to fungal infection and/or enhance the host immune response appear to be logical starting points for the development of new antifungal therapies. Despite the prevalence of fungal infection, its significant morbidity and mortality and the increasing problem of antifungal resistance, antifungal drug development has been under-represented in the development of antimicrobials. The design and development of antifungal therapeutics is, arguably, more complex than the design of antibacterial drugs, as both humans and fungi are eukaryotes and therefore share many common cellular features (55). One of the most obvious differences between fungal and mammalian cells is the cell surface (cell membrane and wall in the case of fungi) and it is perhaps no surprise that the most successful antifungal drugs available today target fungal cell walls (echinocandins) or membranes (azoles, amphotericin B). If we are to design future generations of antifungal drugs, we should look to the immune system as this can readily distinguish between fungi and self and to target fungi for eradication. AMP are one such example of this and are ripe for exploitation as antifungal therapeutic candidates as we discuss in this review (56–59).

## Innate Immunity and Human Fungal Infections

In immunocompetent individuals, innate immunity is the first-line of defence against invasive fungal infection. Host defence peptides (HDP), also termed antimicrobial peptides (AMP), form a key part of the innate immune response to infection and inflammation (60–62). HDP have been found at most sites in the human body, including the oral cavity, skin (including sweat and wound fluid), lungs, blood, tears, gastrointestinal tract, urinary tract & reproductive organs, breast milk and cerebrospinal fluid (63). A number of HDP are produced constitutively by epithelia and this basal level of HDP production can provide a first line of protection against fungal infection. Continuous interactions between fungal pathogen-associated molecular patterns (PAMP) and damage-associated molecular patterns (DAMP) and host pattern recognition receptors (PRR) initiate low levels of NF-κB activation that drives amplified expression of HDP-encoding genes (64–66). Upon greater levels of colonisation, inflammation and/or epithelial damage, expression of HDP genes, and concomitant HDP production, increases significantly (67, 68). For example, human β-defensins, cathelicidin and other HDP are considered integral to the innate immune response to fungal infection in the skin (69), whereas histatins are considered key effectors in the oral cavity (70). In addition to direct antimicrobial activity, HDP can act as immune modulators, for example, by promoting migration of neutrophils and monocytes to the site of infection, by upregulating tumour necrosis factor alpha (TNF-α) production and by chemoattraction of immature dendritic and T cells to modify the adaptive immune response (61, 62, 71). Perhaps unsurprisingly, most studies on the antimicrobial activities of AMP have focused on their antibacterial properties. Most research on antifungal AMP has been directed against *Candida* spp, especially *C. albicans*, with a smaller number of studies assessing activity against *A. fumigatus, Cryptococcus neoformans* and the questionably relevant *S. cerevisiae*. Thus, the direct antifungal activity of HDP, and most other AMP, may be significantly under-realised. In this review we will focus on the direct antifungal activity of AMP and anti-biofilm properties where relevant, but the immunomodulatory properties of HDP/AMP are largely beyond the scope of this manuscript and readers are directed to several excellent reviews on this subject (61, 62, 72–74).

### Histatins

Histatins (Hst) are small histidine-rich HDP with an α-helical conformation in membranes. Histatins, and derivatives, have been investigated for their potential to treat localised infections, including vulvovaginal candidiasis, skin infections, cystic fibrosis lung infections, mucositis and gingivitis/periodontitis (75, 76). First isolated from human parotid saliva, Hst are also found in the saliva of other higher primates. Histatins are secreted by the parotid and submandibular salivary glands. Histatins comprise 12 structurally related members of which Hst-1 and Hst-3 are full-length proteins encoded by two genes, HTN1 (encoding Hst-1) and HTN3 (encoding Hst-3). The smaller proteins, Hst-2 (derived from Hst-1) and Hst-4 to−12 (derived from Hst-3), are generated by proteolytic cleavage of the parent Hst by salivary proteases during secretion (59, 77, 78).

**Figure 1:**
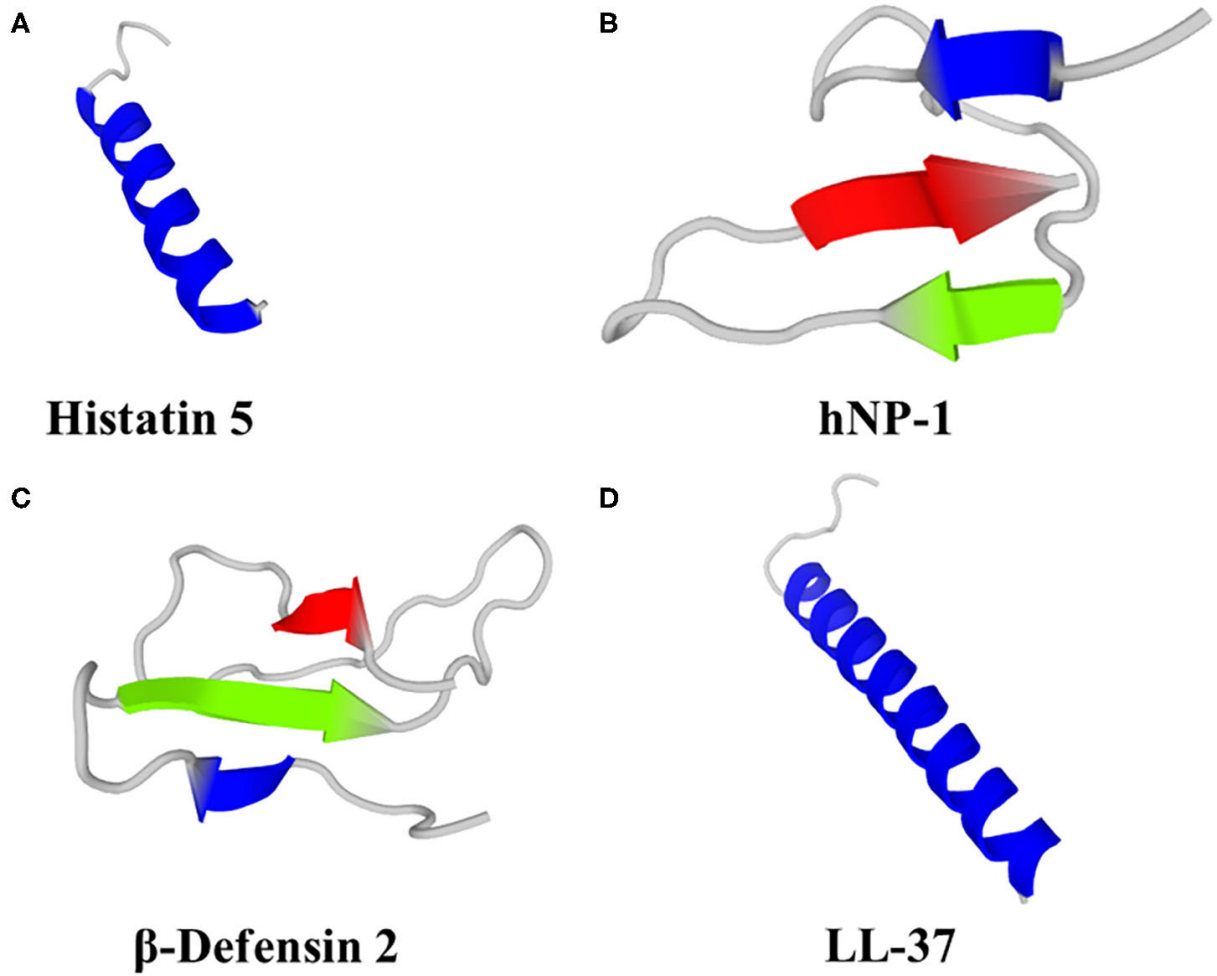
Predicted 3D structures of human HDP; **(A)** Histatin 5, **(B)** Neutrophil Peptide 1 (α-defensin), **(C)** β-Defensin 2 and **(D)** LL-37. Models were generated using PEP-FOLD 3 (79).

Histatins comprise 3 main HDP (Hst -1, -3 and -5), of which Hst-5 (Figure 1A) has the most potent antifungal activity and can be found at concentrations of 15–30 μM in whole saliva (80). Fungicidal activity of Hst has been demonstrated against *Candida* spp. (albeit with little or no activity against *C. glabrata*), *Cryptococcus neoformans* and *A. fumigatus* (70, 81–83). In a study on the efficacy of Hst-5 on *Candida* spp. biofilms, Hst-5 was not effective against planktonic *C. glabrata* (2 isolates; IC_50_ > 100 μM). However, Hst-5 was effective against preformed biofilms of *C. albicans* and *C. glabrata* on poly(methyl methacrylate) discs, resulting in a 50% reduction in biofilm metabolic activity at concentrations of 1.7–62.5 μM (83, 84), albeit less effective than 0.12% chlorhexidine gluconate (84). Hst-1, -3 and -5 can also inhibit germination of *C. albicans* spores, leading to reduced virulence and ability to cause infection (85, 86).

Unlike the membrane-active defensins and cathelicidin, Hst act at multiple levels by mechanisms of action conserved across the Hst family of AMPs. Histatins bind metal ions, including copper, and the presence of Cu improved the antifungal activity of Hst-5 against *C. albicans* (87). In *C. albicans*, Hst-5 binds to fungal cell wall glycans, predominantly β-1,3-glucan (88), and cell wall proteins Ssa1 & Ssa2. Hst-5 is transported into the cell via the fungal polyamine transporters Dur3 and Dur31 in an energy-dependent process (76), and it is the lack of these transporters that forms the basis of the lack of sensitivity of *C. glabrata* to Hst-5 (89). Hst-5 can also be internalized by endocytosis (76) and by direct uptake via interaction with the plasma membrane (90). Hst-5 causes release of K^+^, via the ion transporter Trk1, which causes osmotic imbalance and a consequent loss of cell volume and viability (76, 91). Hst also induce formation of reactive oxygen species (ROS), ATP efflux, inhibition of oxidative phosphorylation and metal ion chelation and these properties could contribute to the fungicidal activity of Hst-5 (76, 92–94). Human saliva also contains other non-immune proteins with antifungal properties, including lactoperoxidase, lactoferrin and lysozyme (95, 96). Interestingly, the antifungal caspofungin (inhibitor β-1,3-glucan biosynthesis) causes a loss of β-1,3-glucans in the *Candida* spp. cell wall, resulting in reduced susceptibility to Hst-5 (88).

Additionally, antibacterial properties of Hst-5 against ESKAPE pathogens have been demonstrated, including anti-biofilm properties (97). Hst may also exert their antimicrobial activities by inhibiting host and microbial proteases and may attenuate tissue damage and microbial propagation during the onset of disease (63). Hst have other functions in the oral cavity, including acceleration of wound healing, tooth enamel mineral homeostasis and pellicle formation (78, 98). Hst-1, -2 and -3, but not Hst-5, can promote re-epithelialization and angiogenesis during wound healing (78) and can prevent the translocation of bacteria across cell layers (99).

### Defensins

There are three distinct families of defensins, α-, β- and θ-defensins which are cationic AMP characterised as three-stranded β-sheet folds stabilised by three conserved and regiospecific disulphide bridges. Humans produce only α- and β-defensins. In addition to their antimicrobial activity, roles in immunomodulation, fertility, development and wound healing have also been indicated (67, 100–102). The immunomodulatory activities of β-defensins include pro-inflammatory responses via recruitment (chemoattraction) of monocytes, macrophages, immature dendritic cells (DC) and T cells to sites of infection/inflammation, thereby providing a link between the innate and adaptive immune system (101, 103–106).

Humans produce six α-defensins; 4 are produced by neutrophils and some other myeloid cells [Human Neutrophil Peptides (HNP) 1–4] and a further two α-defensins (HD-5 and HD-6) are produced by the Paneth cells of the small intestine and some epithelial cells in the reproductive tract (HD-5 only) (101, 107). HNP-1 (Figure 1B) can kill *C. albicans* by depleting intracellular ATP (108) and was fungicidal against *A. fumigatus* (109). HNP-3 demonstrated limited activity against *Cryptococcus neoformans* planktonic cells and biofilms; 72 and 80% survival, respectively, after exposure to 8 μM HNP-3 for 30 min (110). HD-6 prevented adhesion of *C. albicans* to human intestinal epithelial cells, thereby preventing biofilm formation and cell invasion, but not hyphal transition. HD-6 functionality against *C. albicans* is dependent on the self-assembly properties of HD-6 and is non-lethal. HD-6 self-assembles into oligomers, termed nanonets, that entrap pathogens, including *C. albicans*, and prevent them from entering host cells (111).

Humans produce 4 β-defensins (hBD-1 - 4), primarily from epithelial cells that form biological barriers to pathogens at internal-external interfaces of the skin, gastrointestinal tract, respiratory tract and urogenital tract. Computational and bioinformatic approaches suggest at least 28 human β-defensin genes (112). Human β-defensins have direct antimicrobial activity, including via membrane permeabilization, against bacteria, fungi, viruses and unicellular parasites, as well as roles in immunomodulation, reproduction and pigmentation. Human β-defensin 1 is constitutively expressed, whereas hBD-2 (Figure 1C), 3 and -4 are induced in response to various stimuli, including inflammation and infection (101). hBD-1, hBD-2 and hBD-3 killed *C. albicans* by membrane permeabilization (113), hBD-2 was fungicidal against *A. fumigatus* (109) and hBD-3 was fungicidal against *C. glabrata* (114). hBD-1 in reduced form (i.e., lacking disulphide bridges) demonstrated activity against *C. albicans*, unlike the oxidised form, and is found in human colonic mucosa, small intestine crypts and skin epidermis (115). hBD-2 and hBD-3 reduced *C. albicans* adhesion by mediating elevation of Xog1 activity (116). hBD-2 and hBD-9 gene expression was induced by *A. fumigatus* and hBD-2 peptide co-localised with *A. fumigatus* conidia that had been phagocytosed by A549 cells (human alveolar basal epithelial adenocarcinoma cells), but not hyphae (117). Antifungal properties of hBD-1, hBD-2 and hBD-3 have been demonstrated against *C. albicans* (113), including antibiofilm properties of a 15 amino acid fragment from the C-terminus of hBD-3 (118). hBD-1 and hBD-3 were active against *Cryptococcus neoformans* planktonic cells and biofilms, albeit less effective against biofilms (110).

### Cathelicidin

Cathelicidins are cationic HDP containing 12–80 aa (predominantly 23–37 aa) and adopt either α-helix or β-sheet secondary structures in amphipathic helices and include the single human cathelicidin, LL-37 (Figure 1D). The classification of cathelicidins as one family is due to the large evolutionary conserved N-terminal cathelin sequence. However, the highly variable C-terminal region is responsible for most of the broad-spectrum antimicrobial and immunomodulatory activities. Cathelicidin knockout mice were more susceptible to bacterial and viral infection, resulting in a higher morbidity and mortality (119–121). The myriad other properties of cathelicidin have been the subject of several recent reviews (68, 74, 122–124) and are beyond the scope of this manuscript.

The candidacidal activity of LL-37 has been demonstrated in a number of *in vitro* studies (125–129), but activity against other fungi has been demonstrated in a limited number of reports. Antifungal activity of LL-37 was demonstrated against *T. rubrum* (*n* = 2) and *T. mentagrophytes* (*n* = 2) with an MIC/MFC = 12.5–25 μM and was fungicidal against *Malassezia furfur* (25 μM) (130). LL-37 demonstrated antifungal activity (MIC <64 μM) against selected vaginal *Candida* spp. isolates (*C. albicans, C. glabrata, C. krusei* and *C. parapsilosis*), albeit the majority of isolates tested had MIC >64 μM, and was ineffective against preformed *C. albicans* biofilms at ≤32 μM. LL-37 (64 μM) was able to inhibit adhesion of *C. albicans* SC5314 to polystyrene and silicone surfaces, thereby preventing biofilm formation (128). LL-37 associated with the cell wall and/or membrane of *C. albicans* and caused membrane lysis, generation of ROS and release of ATP and other molecules (≤40 kDa) (131). Murine cathelicidin was fungicidal against *Pneumocystis murina* in a dose-dependent manner (10–50 mg/L) (132). The *C. albicans* cell wall β-1,3-exoglucanase, Xog1, interacts directly with LL-37 leading to elevated enzyme activity and subsequent cell wall remodelling and reduced adhesion of *C. albicans* to plastic surfaces (116), oral epidermoid OECM-1 cells and murine urinary bladder at concentrations that were not fungicidal (133). *C. albicans* that did not adhere were aggregated when LL-37 was bound to the cell surface, mediated by preferential binding to cell wall mannans and to a lesser extent chitin and cell wall glucans (133). Secreted aspartyl proteases (SAP1 – 4, 8 & 9) of *C. albicans* were able to hydrolyse LL-37 into smaller peptides *in vitro* and this correlated with a reduction in antifungal and immunomodulatory activity and may facilitate survival of *C. albicans* at sites where LL-37 is produced (134). Interestingly, the *in vitro* growth of *A. fumigatus* and *A. flavus* was stimulated by physiological concentrations of LL-37 (0.97–31.25 mg/L) found in the lung, whereas a scrambled analogue of LL-37 had no such effect (135).

### Other Human Antifungal AMP/HDP

A number of other human AMP/HDP possess documented antifungal activity, including RNases, psoriasin, dermcidin, lactoferricin, antileukprotease/secretory leukocyte protease inhibitor (SLPI), calprotectin, trappin-2/pre-elafin, granulysin, thrombocidins, hepcidins, α-melanocyte stimulating hormone, the chemokine CCL20, substance P, calcitonin gene-related peptide, neuropeptide Y, amyloid β-peptide and vasostatin-1 (136–152).

RNase 3 and RNase 7 demonstrated activity against *C. albicans* (MFC 2.5–5.0 μmol/L) (151), whereas dermcidin demonstrated pH-dependent activity against *C. albicans* with optimal activity at pH 5.5–6.5 (143). SLPI was active against *A. fumigatus*, including spores (137) and *C. albicans* (153). Hepcidins, Hepc20 and Hepc25, inhibited sporulation of *A. fumigatus* and *A. niger* and Hepc20 was fungicidal at 40 μM, whereas both Hepc20 and Hepc25 were only moderately antifungal against *C. albicans* at 30 μM (~1 log kill) (142). Hepc20 was fungicidal against a panel of *C. glabrata* (MIC 60–100 μM), which was enhanced in acidic conditions, whereas Hepc25 was not fungicidal (150, 154). The neuropeptides Substance P, Calcitonin gene-related peptide and Neuropeptide Y demonstrated activity against *C. albicans* (MIC 8.1, 63.1, and 46.5 mg/L, respectively) (146). Lactoferrin and peptides derived from it demonstrated broad-spectrum antifungal activity, including against important pathogenic moulds (e.g., *Aspergillus* spp., *Alternaria* spp., *Fusarium* spp., *Absidia* spp. and dermatophytes) and yeasts (e.g., *Candida* spp., *Cryptococcus* spp. and *Exophiala* spp.) (152). RNase 7, hBD-2 and psoriasin demonstrated activity against dermatophytes, including *T. rubrum, T. mentagrophytes* and *Epidermophyton floccosum*, albeit only psoriasin demonstrated significant activity against *Microsporum canis* (148). Psoriasin demonstrated broad-spectrum antifungal activity with a 90% MIC of ~2 μM against *A. fumigatus, Malassezia furfur, M. canis, Rhizopus oryzae, Saccharomyces cerevisiae, T. rubrum* and *T. mentagrophytes*, but was not active against *C. albicans* at concentrations up to 20 μM (155).

## Novel Antifungal Peptides in Clinical and Preclinical Development

A number of synthetic AMP have been investigated as antifungal therapies (156, 157). AMP with antifungal activity show the same structural diversity as other AMP and include linear and cyclic peptides, lipopeptides and depsipeptides. Over 1100 putative endogenous AMP with antifungal activity have been described (The Antimicrobial Peptide Database; http://aps.unmc.edu/AP/main.php). Antifungal peptides may form α-helices, β-sheets or mixtures thereof and may be cysteine-stabilised. Some are rich in specific amino acids, contain non-natural amino acids or contain non-protein modifications including lipid and carbohydrate moieties. Therapeutic candidate antifungal peptides mostly have a membrane-lytic mechanism of action, but peptides with alternative and even multiple mechanisms of action have been investigated (57–59, 158–161). The structure/composition of fungal cellular membranes vary between species and between yeast and hyphal forms, but in general are more negatively charged than mammalian cell membranes and this may account for the specificity of membrane-active antifungal peptides (58). There have been a number of mechanisms of action both proposed and proven for the interactions of AMP with membranes including the formation of toroidal pores, barrel-stave pores (162), disordered toroidal pores (163), aggregate pores (164), the carpet model (peptide interaction with phospholipid head groups leading to membrane solubilisation) (165). Other less documented mechanisms of action include peptide-induced membrane curvature, induction of cubic lipid phases (166), membrane-thinning/thickening (167), membrane domain formation (168), membrane flip-flop (169), lipid clustering (170) and disruption of membrane potential (171).

### NP213 (Novexatin®)

NP213 is a novel, first-in-class, synthetic AMP therapeutic candidate derived from HDP that was designed specifically as a topical therapy for the treatment of onychomycosis (fungal nail infection) by NovaBiotics Ltd. NP213 is a backbone-cyclised homopolymer of 7 L-arginine residues with a net charge of +7. NP213 is rapidly fungicidal against dermatophytes and other fungi causative of onychomycosis and is more active in the presence of human nail and keratin than in conventional antifungal susceptibility testing (RPMI-1640 liquid medium). NP213 was equally effective against dermatophyte spores and hyphae, unlike terbinafine, which demonstrated limited activity against spores, and demonstrated a 3 log kill within 3–4 h, compared to >24 h for terbinafine. NP213 is membranolytic and dependent on its positive charge for activity. NP213 was efficacious in *ex vivo* models of fungal nail infection, eradicating different *Trichophyton rubrum* isolates after only 28 d application, unlike ciclopirox and amorolfine (172). Preclinical and clinical safety and toxicity testing revealed no systemic exposure following topical application to the skin of mini-pigs or humans (including a maximum exposure study) with no NP213 detectable in plasma. In clinical trials, NP213 was safe and well tolerated. In two randomized, double-blind, placebo-controlled Phase IIa efficacy studies, daily application of NP213 for 28 d demonstrated clearance of infection in 43.3% (after 180 d; trial 1) and 56.5% (after 360 d; trial 2) of patients with mild-to-moderate onychomycosis (determined by culture) (173). NP213 has also been the subject of a Phase IIb study and further clinical studies are planned.

### HXP124

HXP124 is an investigational novel AMP drug candidate in clinical development for the topical treatment of onychomycosis by Hexima Ltd. HXP124 is a novel plant defensin with a cysteine-stabilised αβ-motif structure. HXP124 demonstrated broad-spectrum fungicidal activity against clinically important human pathogens, including *Candida* spp., *Cryptococcus* spp., dermatophytes and other moulds. HXP penetrated human nails and was active in an *ex vivo* model of nail infection. Additionally, HXP124 demonstrated a favourable safety profile in preclinical testing (174). HXP124 has been the subject of a first in human Phase I/IIa trial to evaluate the safety, tolerability and efficacy of daily topical application for 6 weeks in otherwise healthy patients with mild-to-moderate toenail onychomycosis (Australian Clinical Trials ID: ACTRN12618000131257). HXP124 was safe and well-tolerated and substantially reduced the area of infection (>40%) in 15 of 41 patients (37%) analysed after 12 weeks, compared to only 3 of 17 patients (18%) in the vehicle-only group (6 weeks post-treatment) (https://hexima.com.au/).

### CZEN-002

CZEN-002 is a synthetic octapeptide, (CKPV)_2_, derived from α-melanocyte stimulating hormone (α-MSH). α-MSH had previously demonstrated antifungal activity against *C. albicans* (175). CZEN-220 contains the C-terminal tripeptide (KPV) of α-MSH with a Cys-Cys linker to create an octapeptide. CZEN-002 was candidacidal against *C. albicans, C. krusei* and *C. glabrata* at sub-mM concentrations. CZEN-002 is not membranolytic (176). In a rat vaginitis model of *C. albicans* infection, CZEN-002 dose-dependently reduced the number of surviving *C. albicans* over 18 d. CZEN-002 inhibited *C. albicans* phagocytosis by macrophages and inhibited the production of pro-inflammatory cytokines including TNF-α, IL-1β and IL-6, while increasing arginase activity and the secretion of the anti-inflammatory cytokine IL-10, indicating anti-inflammatory properties (177). (CKPV)_2_ exhibited anti-inflammatory effects against human neutrophils (178) and inhibited TNF-α release form endotoxin-stimulated peripheral blood mononuclear cells *in vitro* and *in vivo* (179).

Zengen Inc., developed CZEN-002 for the topical treatment of vulvovaginal candidiasis as an intravaginal gel (56, 180). A phase I/IIa clinical trial reported 88.2% and 87.5% cure (KOH test and culture, respectively) in a total of 18 female VVC patients with VVC that completed the trial in 2004 (https://www.eurekalert.org/pub_releases/2004-05/z-zrp052404.php). A larger dose-ranging Phase IIb clinical trial was planned for 2005 in Canada & EU. The development status of CZEN-002 is not currently known.

### P113

P113 (also known as PAC-113, PAC113 and P-113) is a synthetic amphipathic, α-helical 12 amino acid histatin 5 derivative (AKRHHGYKRKFH) with membrane-permeabilising activity against *Candida* spp. (181) and bacteria (182–185). P113 progressed through clinical development as a topical treatment for oral candidiasis. Complexation with zinc confers greater mechanical stability to the peptide (186). P113 represents the smallest fragment of histatin 5 that retains activity against *Candida* spp. that was comparable to the parent compound. An analogue of P113 containing D-amino acids, P113D, was equally active against *C. albicans*. Substitution of the 3 His residues with Phe or Tyr had little effect on activity against *C. albicans* (MIC 2.2–2.5 mg/L, but substitution of the 2 Arg and 2 Lys residues with Gln abrogated activity (MIC >80 mg/L) (181). P113 was candidacidal against *Candida* spp. (*C*. albicans, *C. tropicalis, C. famata*) in a time- and dose-dependent manner. A series of P113 derivatives have been designed, including a dimer and trimer. P113, P113 dimer and P113 trimer demonstrated limited cytotoxicity against human gingival epithelial cells (LD_50_ > 400 mg/L). The P113 dimer and trimer were more efficacious than P113 against *C. albicans* and *C. krusei* and similarly active against *C. tropicalis, C. dubliniensis* and *C. parapsilosis*, whereas *C. glabrata* was insensitive to all 3 peptides. The P113 trimer retained activity at high sodium acetate concentrations (31.25–93.75 mM), unlike P113 (187). P113, the dimer and the trimer, increased ROS generation and inhibited cellular respiration in *C. albicans* by targeting mitochondrial complex I. This activity was predominantly caused by inhibition of the NADH dehydrogenase in mitochondrial complex I. The P113 dimer and trimer were also able to target an alternative NADH dehydrogenase not present in mitochondrial complex I. The rapid killing by P113, dimer and trimer mostly occurs via ROS generation, rather than depletion of energy (188). In another study, *Candida glabrata* was not sensitive to P113 or other histatins and derivatives (189). As well as evidence for P113 causing membranolysis, similar to the histatins from which it is derived, P113 is rapidly taken up into the cytosol of *Candida* spp. after initial binding to the cell wall, and this process is facilitated by Ssa2p (Heat shock protein 70 (HSP70) chaperone) that can transfer cell wall-bound peptides to membrane permeases to specifically transport peptides into the cytosol. Thus, the antimicrobial activity of P-113 acts through binding to and destabilization of the microbial membrane and through a specific protein receptor on the microbial cell surface (190).

When the His residues at positions 4, 5, and 12 were replaced with the bulky, non-natural amino acids β-naphthylalanine (Nal-P113), salt sensitivity was less pronounced and activity against *Candida* spp. was retained. Such amino acid substitutions may improve activity under physiological salt concentrations (191). P113 was subject to proteolysis by *C. albicans* intracellular enzymes at Ala4 and Lys11, whereas P113D was not (192). Based on studies with Hst-5, the Lys residue at position 8 would be subject to cleavage by *Candida* spp. secreted aspartyl proteases, Sap2 and Sap9 (193). Additionally, histatins (and potentially P113) can form complexes with salivary proteins, e.g., salivary amylase, that can inhibit antifungal activity (194). A possible solution to improve the antifungal efficacy of P113 and other AMP in saliva is to formulate the peptides in delivery systems such as liposomes that facilitate gradual release and limit proteolysis (195). Interestingly, in a rat oral mucosal ulcer model, Nal-P113 increased expression of epidermal growth factor (EGF) and fibroblast growth factor-2 (FGF-2) and decreased the expression of transforming growth factor-β1 (TGF-β1), whereas in an *in vitro* wound healing assay, Nal-P113 promoted migration of human immortalized oral epithelial cells, indicating that application of Nal-P113 might be an effective therapeutic approach for recurrent aphthous stomatitis (196).

General Biologicals Corporation (GBC) currently market P113-containing compounds as part of their over-the-counter antibacterial “oh-care” range Whilst apparently continuing development of P113 for the treatment of oral candidiasis. A Phase I/IIa clinical trial demonstrated that P113 as an oral mouthrinse was generally safe and well-tolerated and similarly efficacious in curing oral candidiasis as the gold standard, as 37% of PAC-113 patients were assessed as clinically cured at day 14 compared to 36% of Nystatin patients (56, 180). A randomized, examiner-blinded, positive-controlled, parallel design Phase IIb clinical trial of PAC113 oral mouth rinse was carried out in 2008 in 223 HIV seropositive individuals with oral candidiasis and included 3 different concentrations of PAC113 (0.15, 0.075, and 0.0375%) compared to Nystatin oral suspension to determine whether there was elimination or a reduction in clinical signs and symptoms of oral candidiasis. Unfortunately, no results were posted for this trial (ClinicalTrials.gov Identifier: NCT00659971). In a double-blinded, randomized, controlled clinical trial to evaluate the safety and toxicity of three histatin (P-113) concentrations in gel formulations, and to assess potential clinical benefit on the development of gingivitis, 106 healthy subjects without gingivitis were enrolled. All formulations were safe and well-tolerated and efficacy data revealed that P113 gels applied twice daily may reduce experimental gingivitis in humans (197). In another phase 2 multi-centre clinical study, a P113 mouth rinse was safe and well-tolerated and reduced the development of gingivitis in 294 healthy subjects using the formulation twice daily in place normal oral hygiene procedures (198). In a double-blinded, randomized clinical study, 37 patients with moderate or severe chronic periodontitis were treated on one tooth with 20 mg/L Nal-P113 or placebo on days 0 and 3 and on day 7 teeth were sampled. Treatment with Nal-P113 improved periodontal clinical status, reduced plaque/biofilm formation compared to controls (199).

### Omiganan

Omiganan (MX-226 or MBI-226) is a synthetic AMP (ILRWPWWPWRRK-amide) derived from indolicidin, originally isolated from bovine neutrophils, with antifungal (200, 201), antibacterial (200, 202), anti-biofilm (203, 204), antiviral (205) and immunomodulatory properties (206). Omiganan was active against *Candida* spp.; *C. albicans* (MIC 32–>512 mg/L; *n* = 104), *C. glabrata* (MIC 128–>512 mg/L; *n* = 27), *C. krusei* (MIC 16–256 mg/L; *n* = 26), *C. parapsilosis* (MIC 32–256 mg/L; *n* = 30) and *C. tropicalis* (MIC 8–64 mg/L; *n* = 27) (200) and moulds, including *Aspergillus* spp. (MIC 16–1,024 mg/L; *n* = 10), *Curvularia* spp., *Fusarium* spp., *Paecilomyces variotii* and *Penicillium* spp. (MIC 1–256 mg/L; *n* = 10) (201). 100 mg/L omiganan caused a 1–2 log kill against *C. albicans* (*n* = 3) within 1 h exposure (129). Interestingly, an omiganan analogue with the sequence reversed (KRRWPWWPWRLI-NH_2_) was more active against *C. albicans* (Forward MIC 128 mg/L; Reverse MIC 32–64 mg/L) and both were equally effective against *A. niger* ATCC16404 (MIC 64 mg/L) (207). An all D-enantiomer analogue of omiganan demonstrated the same antimicrobial activity as L-omiganan, but was less susceptible to skin proteases (t1/2 >120 min and t1/2 = 10 min, respectively) (208). In an *ex vivo* pig skin infection model, ≥0.1% (w/w) omiganan (in an aqueous gel) was active against *C. albicans* ATCC14053, causing a 2–3 log kill after 24 h, whereas in an *in vivo* guinea pig skin infection model 1% (w/w) omiganan cased a 2 log kill after 24 h (209).

Omiganan has been the subject of 16 clinical trials in the US and 10 in Europe (ClinicalTrials.gov clinicaltrialsregister.eu), probably making it the most studied AMP in humans, albeit all trials were for topical application, including acne vulgaris, rosacea and sebhorrhoeic dermatitis. Unfortunately, none of these clinical trials investigated the antifungal properties of omiganan, although one trial into the use of omiganan for the prevention of central venous catheter-related bloodstream infections described that they would test for fungaemia, bacteraemia and sepsis (ClinicalTrials.gov Identifier: NCT00027248). Unfortunately, no results for this study, sponsored by BioWest Therapeutics Inc, have been posted. In a later Phase III study of 1,859 hospitalised patients, omiganan 1% gel was compared to 10% povidone-iodine for the prevention of catheter infection/colonisation in patients with central venous catheters, but results were disappointing and the trial failed to achieve its primary efficacy end-point of reducing local catheter site infections (ClinicalTrials.gov Identifier: NCT00231153) (56, 180, 209). In two recent Phase II clinical trials sponsored by Cutanea Life Sciences (EudraCT Number: 2015-002724-16 & 2015-005553-13), the safety and efficacy of omiganan in the treatment of human papillomavirus-induced genital lesions (*n* = 12) or external ano-genital warts (*n* = 24) was assessed. Omiganan was safe and well tolerated by all patients. Human papillomavirus load significantly reduced after 12 weeks of treatment with omiganan compared to placebo, but only in the external ano-genital warts patients (205). Whilst clinical development of omiganan appears to be ongoing, omiganan has been proven to be safe and generally well-tolerated as a topical antimicrobial. Its efficacy has yet to be proven in the clinic. Carefully designed trials with appropriate efficacy/outcome measures and application of the peptide in appropriate formulations will be critical to ensure success and potential translation of this compound's promising *in vitro* antifungal data.

### hLF1-11

The AMP hLF1-11 (GRRRRSVQWCA) comprises the first 11 amino acids of human lactoferrin and is a multi-functional peptide with antibacterial activity (185, 210, 211), antifungal activity (212) and immunomodulatory properties (213, 214). hLF1-11 demonstrated antifungal activity *in vitro* against *C. albicans* (MIC 22–44 mg/L; *n* = 11), including oral and vaginal isolates (212, 215). Pre-treatment of fluconazole-resistant *C. albicans* with non-candidacidal concentrations of hLF1-11 (4–8 μM) was synergistic with fluconazole, rendering this strain fluconazole sensitive. The combination of hLF1-11 and fluconazole was also effective against *C. glabrata, C. krusei, C. tropicalis* and *C. parapsilosis* (216). hLF1-11 caused mitochondrial calcium uptake which stimulated an increase in mitochondrial membrane potential and permeability, resulting in the synthesis and secretion of ATP and ROS production, leading to *C. albicans* cell death (217). hLF1-11 was also active against *A. fumigatus* hyphae (EC_50_ 29 ± 5 μM) and spores (MIC 5 ± 4 μM) (218).

hLF1-11 (88–176 mg/L) prevented *C. albicans* biofilm formation with almost complete inhibition of metabolic activity, a 2 log reduction in cell viability (176 mg/L) and decreased expression of selected biofilm-associated genes. However, hLF1-11 demonstrated poor activity against pre-formed biofilms (215). hLF1-11 (88–176 mg/L) prevented *C. parapsilosis* (*n* = 3) biofilm formation and 55 mg/L hLF1-11 significantly reduced the amount of biofilm formed. When *C. parapsilosis* CP7 was allowed to adhere to the surface of 96-well plates or peripheral Teflon catheter pieces for 1.5 or 3 h, hLF1-11 (≥44 mg/L) significantly reduced the amount of biofilm formed and metabolic activity, whereas after being allowed to adhere for 6 h, 44 mg/L hLF1-11 was ineffective at preventing adhered cells developing into biofilms and both 44 and 88 mg/L hLF1-11 were ineffective when *C. parapsilosis* had been allowed to adhere for 24 h. Incubation of *C. parapsilosis* CP7 with 44 mg/L hLF1-11 led to reduced expression of the adhesin gene CpALS7, the biofilm formation-associated gene *CpACE2* and the β-glucan synthase catalytic sub-unit 1 gene *CpFSK1* (219). Coating of hLF1-11 onto titanium surfaces by atom transfer radical polymerization reduced adhesion of *Streptococcus sanguinis, Lactobacillus salivarius* and a mixed microflora derived from human dental plaque (220), whereas attachment of hLF1-11 to chitosan films via the cysteine residue increased the adhesion of *Staphylococcus aureus* ATCC33591 to the film, albeit with some reduction in viability (221). Thus, hLF1-11 may have application in prevention of infection of implanted medical devices provided careful consideration is given to the manner of surface attachment. hLF1-11 was not haemolytic at concentrations up to 200 mg/L and caused no significant loss of viability of murine osteoblast MC3T3-e1 cells at a concentration of 400 mg/L (222).

hLF1-11 demonstrated synergistic inhibition of *C. albicans* SC5314 in combination with caspofungin *in vitro*. When tested in the *Galleria mellonella* (wax moth) larva model of infection hLF1-11 was not toxic (≤100 mg/kg), but these concentrations were not effective at improving survival in larvae infected with *C. albicans* (2.8–3.0 × 10^5^ cfu inoculum) and in this model, the combination of 25 mg/kg hLF-11 and 0.5 mg/kg caspofungin also resulted in no enhanced survival (223). In a neutropenic murine model of systemic candidiasis (established for 24 h) with a fluconazole-resistant *C. albicans* isolate 0.4 μg/kg hLF1-11 caused a ~1.5 log reduction in *C. albicans* kidney burden after 18 h and mice treated with up to 40 μg/kg hLF1-11 had smaller and fewer infectious foci in their kidneys and grew predominantly as yeast, unlike the hyphal growth observed in the kidneys of untreated mice. hLF1-11 was also able to inhibit the yeast-hyphal transition *in vitro* (217).

Exposure of monocytes to hLF1-11 during GM-CSF-driven differentiation is sufficient to direct differentiation of monocytes toward a macrophage subset characterized by both pro- and anti-inflammatory cytokine production (IL-10 and TNF-α) when subsequently exposed to heat-killed *C. albicans* and these macrophages also demonstrated increased responsiveness to bacterial lipopolysaccharide (LPS), lipoteichoic acid (LTA) and heat-killed *C. albicans* (213). Following intracellular uptake by monocytes, hLF1-11 bound to myeloperoxidase (MPO) and inhibited the chlorination and peroxidation activity of MPO (224). hLF1-11 also facilitated differentiation of human monocytes to dendritic cells (DC) with increased expression of HLA class II antigens and dectin-1 (a *C. albicans* PRR) and increased phagocytosis of *C. albicans*, but not *Staphylococcus aureus*. Upon stimulation with *C. albicans*, hLF1-11-differentiated DC produced increased amounts of ROS and the cytokines IL-6 and IL-10, but not IL-12p40 or TNF-α. Supernatants from hLF1–11-differentiated DCs caused CD4+ T cells to produce increased concentrations of IL-17, but reduced IFN-γ, following stimulation with *C. albicans* (214).

hLF1-11 has been the subject of 4 proposed human trials, sponsored by AM-Pharma, and registered on ClinicalTrials.gov although only one was completed. The completed trial was to determine the safety of a single intravenous dose of hLF1-11 (5 mg, single dose IV) in 8 autologous haematopoietic stem cell transplant recipients (HSCT) (ClinicalTrials.gov Identifier: NCT00509938). The safety and tolerability of hLF1-11 had to be established in HSCT recipients as they are at risk of developing, but have not yet developed, infectious complications due to invasive fungal disease. An earlier study in 48 healthy volunteers (36 hLF1-11 and 12 placebo) had established that single ascending intravenous doses (0.005–5 mg, single dose IV) and multiple intravenous doses (0.5 & 5 mg, single dose IV) were safe and well tolerated. HSCT patients differ from healthy volunteers as they have received myeloablative treatment which arrests haematopoiesis, resulting in neutropenia, but also causes mucosal barrier injury. Both of these predispose HSCT patients to fungal infections which typically occur during the week after transplant. It was therefore essential to know that hLF 1-11 is safe when given during neutropenia and mucosal barrier injury before infections ensue. A single 5 mg (single dose IV) dose was well-tolerated in patients with a side effect of elevated liver enzymes (alanine aminotransferase and aspartate aminotransferase) that was reversible and may have been related to treatment (225). A further study to determine the effect of multiple doses of hLF1-11 in HSCT patients (ClinicalTrials.gov Identifier: NCT00430469) was withdrawn by the sponsor prior to patient recruitment. Another of the withdrawn studies, one was a phase IIa, double-blind, randomized study to determine the tolerability and efficacy of hLF1-11 in patients with proven candidemia with concomitant fluconazole treatment (ClinicalTrials.gov Identifier: NCT00509834), but unfortunately the target patient population was not available. It is clear that hLF1-11 is generally safe and well-tolerated in healthy subjects and HSCT patients at the dose ranges tested thus far and that this peptide has *in vitro* and preclinical efficacy in models of fungal infection. It remains to be seen how effective this peptide can be in clinical use.

### Iseganan (IB-367)

Iseganan (IB-367) is a synthetic AMP containing 17 amino acid residues derived from protegrin I, part of the cathelicidin family of AMP, that has been in clinical development for the treatment of oral mucositis (226–228) and ventilator-associated pneumonia (229). Iseganan was selected as the most promising candidate for the prevention of oral mucositis based on a study of structure–activity relationships of synthetic protegrin analogues (230). Iseganan demonstrated antibacterial activity (231, 232), antifungal activity (233, 234), anti-parasitic (235), anti-biofilm activity (236) and both antibacterial and anti-endotoxin activity in rat models of septic shock (237). Iseganan was fungicidal against dermatophytes (MIC 8–16 mg/L (*n* = 20; MFC 16–32 mg/L (*n* = 20) (234). and *C. albicans* (MIC 4–8 mg/L; MFC 4–32 mg/L (*n* = 5), *C. glabrata* (MIC 2–16 mg/L; MFC 2–16 mg/L (*n* = 5), *C. parapsilosis* (MIC 8–32 mg/L; MFC 16–>128 mg/L (*n* = 5), *C. krusei* (MIC 4–16 mg/L; MFC 4–64 mg/L (*n* = 5) and *C. tropicalis* (MIC 2–4 mg/L; MFC 2–4 mg/L (*n* = 5) (233), although activity against *A. fumigatus* ATCC16404 was poor (MIC/MFC = 256 mg/L) (238). Local application of Iseganan (IB-367) reduced mucositis severity in a hamster model of oral mucositis which correlated with a >100-fold reduction in oral microflora densities in a dose-dependent manner (239).

A multi-centre double-blind, placebo-controlled Phase III trial to determine the efficacy of Iseganan HCl rinse in reducing the severity of oral mucositis in 323 patients (163 iseganan and 160 placebo) receiving stomatotoxic chemotherapy (PROMPT-CT trial). Iseganan (9 mg in 3 ml) was administered as a swish and swallow solution, six times daily for 21–28 d and was safe and well-tolerated. In this study, 43 and 33% of Iseganan and placebo patients, respectively, did not develop ulcerative oral mucositis. Iseganan patients experienced less mouth pain, throat pain and difficulty swallowing compared to placebo patients and experienced lower stomatitis scores (226). However, other studies failed to demonstrate a benefit of Iseganan in causing reduction in oral mucositis (227, 228). Stomatotoxic chemotherapy can induce changes in the oral microflora that may cause oral and systemic infections in myelosuppressed cancer patients and studies suggest that reduction of the microbial load in the oral cavity has some clinical benefit. A sub-analysis of the first trial was conducted to assess the antimicrobial activity of Iseganan in this patient population. Microbial cultures were generated before and after the daily Iseganan mouth rinse. Iseganan significantly reduced total microbial load in the oral cavity, mainly due to decreased numbers of streptococci and yeasts. This antifungal activity is of interest as oropharyngeal candidiasis is common in immunocompromised patients and some elderly populations (240). A multinational, double-blind, randomized, placebo-controlled trial of Iseganan (371 patients) applied topically to the oral cavity vs. placebo (354 patients) in intubated patients receiving mechanical ventilation for up to 14 d was conducted to determine the occurrence of microbiologically confirmed ventilator-associated bacterial pneumonia (VAP) measured among survivors up through Day 14 (ClinicalTrials.gov Identifier: NCT00118781). The peptide was deemed to be safe and well tolerated but the study's primary efficacy end-points were not met [no significant differences in the rate of VAP among survivors between patients treated with Iseganan (16%) and those treated with placebo (20%; *p* = 0.145) (229)]. The design of the study was potentially flawed due to the short exposure time of Iseganan to potential pathogens (241). Thus, as a proven safe and well tolerated candidate when applied topically in very sick patients with preclinical antifungal activity, Iseganan has the potential to be developed as an AMP for the treatment of oropharyngeal candidiasis and for topical application for the treatment of other fungal infections.

### LTX-109

LTX-109 (LTX109, Lytixar, LTX 109) is an AMP peptidomimetic (Arg-Tbt-Arg-NH-EtPh) that was in clinical development by Lytix Biopharma AS. LTX-109 contains 2 arginine residues, a central modified tryptophan residue (2,5,7-tri(*tert*-butyl)tryptophan) and an ethylphenyl group at the C-terminus (242) with antibacterial (243, 244) and antifungal activity (245). LTX-109 was fungicidal against *S. cerevisiae* (MIC 8 mg/L), causing a 3 log kill within 60 min, and was also active against pre-formed *S. cerevisiae* biofilms. LTX-109 disrupted *S. cerevisiae* membrane integrity by a sphingolipid-dependent mechanism (245).

Topical LTX-109 has been the subject of 3 clinical trials in Gram-positive bacterial infections; nasal decolonisation of *Staphylococcus aureus* (Clinical trials identifier: NCT01158235), a role in non-bullous impetigo (Clinical trials identifier: NCT01803035) and Gram positive skin infections including patients with mild eczema/dermatoses such as atopic dermatitis (Clinical trials identifier: NCT01223222). The study for nasal decolonisation of *Staphylococcus aureus* was a randomized, double-blind, dose escalation phase I/IIa study conducted at a single centre to compare the efficacy, safety, tolerability, bioavailability and efficacy of 3 days nasal treatment with LTX-109 (TID) applied directly to the anterior nares vs. vehicle in persistent nasal carriers of *Staphylococcus aureus*. LTX-109 was safe and well-tolerated and treatment with LTX-109 resulted in a reduction in *Staphylococcus aureus* counts after only 1 day of application. A significant reduction of the number of CFU below the detection limit compared to the vehicle group was demonstrated in subjects treated with 2 and 5% LTX-109 after 2 days of treatment. The most frequently reported AEs related to the application site were itching, burning, pain, and redness (*n* = 26) and the subjects in the 2 and 5% LTX-109 treatment groups reported more of these symptoms than did the 1% or vehicle groups (246). Unfortunately, no results are available for the other 2 LTX-109 clinical trials. Given the positive clinical safety and tolerability data following topical application over multiple days in bacterial infection, together with promising antifungal activity *in vitro*, LTX-109 could be a promising candidate for the treatment of fungal infection.

## Antibiofilm Peptides

The ability of fungi to form biofilms have been associated with high rates of morbidity and mortality, yet compared to bacterial biofilms and bacterial anti-biofilm compounds, the field of fungal biofilm research remains in its infancy. Fungal biofilms consist of adherent cells (on biotic or abiotic surfaces) surrounded by an extracellular matrix which can reduce antifungal efficacy and impair immune responses (247, 248). In addition to direct antifungal activity some AMP/HDP, *in vitro*, can prevent biofilm formation and/or eradicate preformed biofilms via mechanisms associated with fungal adhesion, cell wall perturbation, generation of ROS and gene regulation (59, 249). Although not yet in clinical use, the search for AMP with “druggable” antibiofilm properties remains ongoing (56, 250). For example, in the case of *Cryptococcus neoformans* biofilms, formation is dependent on the production of the polysaccharide capsule (251). hBD-1 and hBD-3 were active against *Cryptococcus neoformans* planktonic cells and biofilms, albeit less effective against biofilms (110), whereas lactoferrin was not effective against *Cryptococcus neoformans* biofilms (251). Hst-5 was effective against planktonic *C. albicans* (IC_50_ 2.6–4.8 μM; *n* = 3), but not *C. glabrata* (IC_50_ > 100 μM; *n* = 2). However, Hst-5 was active against preformed biofilms of *C. albicans* and *C. glabrata* on poly(methyl methacrylate) discs, resulting in a 50% reduction in biofilm metabolic activity at concentrations of 1.7–6.9 μM (*C. albicans*; *n* = 3) and 31.2–62.5 μM (*C. glabrata*; *n* = 2) (83). LL-37 was able to prevent *C. albicans* biofilm formation on silicone elastomer discs (used in the manufacture of medical devices) at sub-MIC concentrations without a concomitant reduction in *C. albicans* viability, whereas LL-37 had no effect on pre-formed *C. albicans* biofilms (128). Thus, AMP have promise as anti-biofilm agents against fungi as well as bacteria.

## Future Directions for Antifungal Peptide Design and Development

### Antifungal Peptides in Preclinical Development

A limited number of AMP are in preclinical development for the treatment of fungal infections and have been extensively reviewed (156, 157, 252–254). In this section we will provide a non-exhaustive review of some of the later stage preclinical antifungal AMP candidates likely to be closer to clinical testing.

NP339 is a preclinical drug candidate being developed as an intravenous therapy for life threatening invasive fungal disease (bloodstream and deep tissue fungal infections) including those caused by yeasts and moulds that are resistant to existing antifungal therapies. An inhaled form of NP339 is also under development for direct delivery into the airways in patients with, or at risk of respiratory fungal infections, including Allergic Bronchial Pulmonary Aspergillosis (ABPA) and pulmonary fungal infections in cystic fibrosis patients. NP339 is a synthetic 2 kDa cationic linear AMP that has been engineered from β-defensins.

NP339 targets the fungal membrane and kills fungi by membrane disruption and cell lysis. This mechanism of action is specific to fungal cells and NP339 is not cytotoxic at significantly higher concentrations than are required to achieve antifungal activity. NP339 kills more rapidly than conventional classes of antifungals, including against metabolically active and inactive fungi and is also sporicidal. NP339 is active against a broad range of clinically relevant fungal pathogens, including *Aspergillus* spp., *Candida* spp. and *Cryptococcus* spp., as well as emerging fungal pathogens including Mucorales, *Scedosporium* spp. and *Exophiala* spp. (255). Nebulised NP339 as a monotherapy, or in combination with amphotericin B, elicited a reduction in lung burden relative to vehicle in murine models of invasive pulmonary aspergillosis (256).

In addition to P113 (see Section P113), Demegen had a second AMP, D2A21, in pre-clinical development (257). D2A21 was a synthetic peptide derived from cecropin (258) being investigated for a number of antimicrobial indications and was formulated as a topical gel (Demegel). D2A21 demonstrated *in vitro* antifungal activity against *C. albicans, A. niger, Mucor* spp. and *T. mentagrophytes*, as well as antibacterial, antiparasitic and potential anti-tumorigenic activity (257). Potential antimicrobial indications include fungal infections, sexually-transmitted infections caused by *Chlamydia trachomatis* (259), and *Trichomonas vaginalis* (260) (for which *in vitro* activity was demonstrated) and burn wound infections (261, 262), In an *in vivo* infected burn wound model in Wistar rats, D2A21 demonstrated significant antibacterial activity against *P. aeruginosa* infection, sterilized burn eschar and decreased the bacterial load in subeschar, leading to significantly improved survival (261, 262).

ETD151 is a preclinical AMP drug candidate derived from ARD1 (a heliomycin peptide), a naturally occurring AMP from the lepidopteran *Heliothis virescens* (tobacco budworm). ETD151, developed by EntoMed SA, is a 44 aa AMP intended for the treatment of serious invasive fungal infections affecting immunocompromised patients (263). ETD151 was derived from ARD1 by site-directed mutagenesis, following recombinant expression in *Saccharomyces cerevisiae* to create a peptide with increased cationicity (264). ETD151 demonstrated promising antifungal activity *in vitro* (MIC_50_ 0.1–6.25 mg/L against *C. albicans, Cryptococcus neoformans, A. fumigatus, F. solani* and *Scedosporium prolificans*) (264). In murine models of systemic *C. albicans* or *A. fumigatus* infection, EDT-151 was effective when compared to amphotericin B and azoles and was non-toxic following intravenous administration (263). ETD151 has yet to enter clinical trials to the knowledge of the authors, however, most recently, the antifungal activity of ETD151 has been assessed against *Botrytis cinerea*, a necrotrophic plant pathogen responsible for gray mold disease, for use as a fungicide in crop protection (265).

### Preclinical Activity Testing

Antimicrobial peptides, whether antifungal, antibacterial, antiparasitic or antiviral, cannot be developed through the same preclinical and clinical pathways as small molecule drugs. We cannot assume or expect that methods for determining antimicrobial activity that are employed in the development of antibiotics and other “small” molecule antimicrobials will be appropriate for the development of AMP as drug candidates. We say “small” as the authors acknowledge that many clinically used antimicrobials do not obey the traditional definition of small, i.e., <500 Da, from Lipinski's rule of five (266), but are nevertheless generally smaller than most AMP. Evaluation of AMP as antimicrobial drug candidates begins with *in vitro* antimicrobial susceptibility testing in which a number of key parameters need to be taken into consideration, including media composition, growth phase, oxygen, temperature and other biological matrices (Table 2) (267, 268). This also applies to *in vitro* cytotoxicity testing (269, 270), formulation and delivery considerations (see Section Formulation and Delivery) and the choice of models for *in vivo* testing (271). It is probable that with adequate consideration given to the factors outlined above and also appropriately designed clinical trials there would be significantly more AMP in preclinical and clinical development and the importance of this is described in detail in a new review of the subject (268).

**Table 2:** Factors influencing preclinical antimicrobial activity testing of AMP.

***In vitro***	***Ex vivo***
pH & ionic strength	Biological matrices (e.g., blood)
Temperature	Mammalian cells
Medium type/composition	Intracellular pathogens
Nutrient concentrations	
Buffer	
Bicarbonate	
Metal ions	
Salt (NaCl)	
Polysorbate-80	
Synergy/Antagonism with other antimicrobials	
Inoculum size	
Growth Phase (e.g., biofilms, persisters, spores, small colony variants and other phenotypic variants)	
Charge effects	
Solubility	
Laboratory materials	
Proteolysis	
Biological macromolecules (e.g., protein, DNA)	
Oxygen (hyper-, norm- & hypoxia)	
Mono/Polymicrobial interactions	

### Rational Drug Design

As stated above, most manuscripts describing AMP &/or peptidomimetics focus on antibacterial properties, but when considering the AMP themselves, and not their target, most reports focus on the isolation of AMP from increasingly unusual organisms (272–274), library screening (275–277) and attempting to identify or modify AMP to have the highest possible level of antimicrobial activity (i.e., lowest MIC). This is perhaps reflected by the fact that The Antimicrobial Peptide Database (http://aps.unmc.edu/AP/main.php) now contains over 3100 entries. Despite this, no AMP has achieved approval by the regulatory authorities as an antimicrobial therapeutic in clinical practice.

Whilst our understanding of the biology and function of AMP remains incomplete, especially how peptides behave in complex biological systems, we are gaining sufficient insight that researchers are increasingly making use of this biological knowledge and even computational approaches to design novel, synthetic AMP (278–282). Novel, informed drug-design approaches to identify AMP is aided by the vast sequence space available (78, 283). Other approaches have taken known host defence peptides and attempted to optimise them using a variety of approaches (281, 284–286).

At a less complex level, rational drug design principles can be applied to designing AMP to target specific pathogens at specific anatomical sites. As described above, NP213 has completed Phase II clinical trials for the treatment of onychomycosis (173). NP213 was designed at the outset as an antifungal peptide, but one that also needed to have specific physicochemical properties that would facilitate penetration into human nail (172). Human nail is a highly effective biological barrier and delivery of therapeutics to the nail and nail bed is challenging (287, 288). Additionally, keratin, the major constituent of the nail, binds to and inactivates many of the existing small molecule antifungal classes, thus compromising therapy (289). AMP/HDP are expressed and produced in the nail (290–293) and several HDP/AMP are antifungal against dermatophytes, including LL-37 (130), hBD-2, RNase7 and Psoriasin (148). AMP therefore constituted a logical starting point for the design of a novel therapeutic for the treatment of onychomycosis. NP213 is highly hydrophilic and positively charged (net charge +7), properties that should facilitate nail penetration as the nail is a negatively-charged, concentrated hydrogel under physiological conditions (294). Additionally, NP213 is small compared to most AMP/HDP (7 aa vs. ~12–>50 aa) that are already known to penetrate nail (292, 293) and this low molecular weight should also facilitate nail penetration (295). One of the known drawbacks of peptide/protein therapeutic candidates is susceptibility to hydrolysis, especially proteolysis (296), which is of especial concern with respect to dermatophytes as they are known to produce multiple classes of proteases/peptidases that enable them to hydrolyse keratin (297, 298). NP213 is a cyclic peptide and therefore not prone to hydrolysis by exoproteases and the limited sequence diversity within NP213 limits the classes of endoproteases that could hydrolyse NP213 (https://www.ebi.ac.uk/merops). Therefore, even prior to peptide synthesis, NP213 had been designed to function as an antifungal at this unique site of infection.

### Formulation and Delivery

In comparison to the considerable body of research focusing on the discovery of AMP and the optimisation of their activity, considerably less effort has been given to delivery systems, formulation or routes of administration for AMP. Formulation and delivery of AMP will play key roles in efficacy outcomes including reducing degradation of protease-susceptible AMP, limiting binding to plasma and other proteins and macromolecules, controlling dose-exposure parameters and even potentially targeting pathogens directly (e.g., intracellular pathogens or pathogens in biofilms). This topic merits a separate manuscript and several excellent reviews have already been written to that end (299–303).

As has been published widely, an issue for the development of certain peptide therapeutics is the potential for proteolysis, whether by proteases of host or microbial origin (304, 305). Infected tissue is often characterised by high levels of proteases, both microbially- and host-derived (306). Possible solutions to the problem of proteolysis include formulation of the peptide to afford protection from proteases, including liposomal formulations, as used for other drugs (307), use of non-natural or D-enantiomer amino acids (308, 309), design and development of peptidomimetics (310, 311) and multivalent peptides (312).

When considering formulation of AMP, the characteristics of both the AMP and the carrier require consideration. AMP charge (and its type and distribution), size, solubility, hydrophobicity and structure can affect loading and activity, as can the properties of the carrier including charge, pH, ionic strength, pore/mesh size, conjugation method (where appropriate). Formulation and delivery approaches that have been tested for AMP include the use of hydrogels (313–315), liposomal formulations (195, 316), carbon nanotubes (317), PEGylation (270, 299) and nanoparticles (318, 319). Appropriate formulation and delivery strategies may also allow us to resurrect and re-investigate some of the candidate AMP therapies that have previously been abandoned because *in vivo* and/or clinical efficacy was significantly diminished vs. *in vitro* data.

## Other Antifungal Immunotherapeutics

The antifungal properties of endogenous HDP are such that these peptides are obvious templates for the design and development of synthetic therapeutic antifungal AMP. As described in preceding sections of this review, AMP have shown early promise as therapeutic candidates. The optimal clinical pathway (trial design, endpoints, formulation etc.) to demonstrate translation of their therapeutic potential into clinical use may not have been carved out as yet however, to the detriment of a number of molecules no longer in development as a result. There are, however, other potential immunotherapeutics that could be deployed alongside antifungal AMP and even existing classes of antifungal therapy; in each case to further enhance infection resolution and eradication. In particular, invasive (systemic) fungal infections predominantly affect immunocompromised patients and there are potential benefits in strengthening those aspects of the immune response that remain functional in these individuals in order to combat systemic fungal infection (30, 320, 321). In cases of invasive aspergillosis or systemic candidiasis, clinical practice guidelines recommend reduction or reversal of immune suppression (31, 322, 323), but in many cases this is simply not feasible due to the initial pathology in cases of stem cell malignancy. In some cases, the reversal of immune suppression can result in immune reconstitution inflammatory syndrome (IRIS), causing increased morbidity and mortality due to “cytokine storm” and an exaggerated host inflammatory response (324, 325). Identifying patients, therefore, for whom particular antifungal immunotherapies are appropriate is critical. It is essential to avoid overtly “boosting” any aspect of the host response in patients who are not entirely immunodeficient in order to mitigate potential immunotoxicity or hyperinflammation. Directly acting antifungal AMP with no host cell pharmacology are potentially the class of immunotherapy with broadest cross-patient applicability for fungal disease in this context. The development of biomarkers to predict responses to antifungal immunotherapy may be beneficial for broader, future adoption of fungal immunotherapy (326) and clinical trial design for these treatments will also require careful consideration as potential patient pools are likely to be limited compared to oncology trials where immunotherapeutics are more commonly used.

Adjunct immunotherapy strategies include the adoptive transfer of activated immune cells with antifungal activity, the administration of immune-activating cytokines in combination with antifungal therapy or the use of antibody therapy. Other approaches being studied include transfusion of leukocytes pre-loaded with antifungals, modulated T cells (e.g., stimulated *ex vivo* and re-infused) and investigation of potential vaccine strategies (321, 327–332). Some of these approaches will be described in subsequent sections.

### Immunostimulatory Molecules

#### Interferon-γ

A number of clinical studies have demonstrated beneficial effects of recombinant interferon-γ (IFN-γ) administration in combination with antifungal therapy in immunocompromised patients with systemic fungal infections, including *Candida* spp. and *Aspergillus* spp. infection (*n* = 8 patients) (333), chronic granulomatous disease (CGD) (*n* = 130) (334–336), HIV infection (*n* = 173) (337–339), leukaemia (*n* = 5) (340, 341), and transplant patients (*n* = 7) (342), in a single patient with *S. aureus* liver abscess and invasive *C. albicans* infection (343), in a single patient with intracerebral aspergillosis (344), in two patients with progressive chronic pulmonary aspergillosis (345), and in two patients with idiopathic CD4 lymphopenia and cryptococcal meningitis (346). In the study of Delsing and co-workers, rIFN-γ administration partially restored immune function as evidenced by increased production of proinflammatory cytokines involved in antifungal defence by leukocytes (IL-1β, TNFα, IL-17, and IL-22) and increased human leukocyte antigen DR (HLA-DR) positive monocyte production in patients where levels were low (333). IFN-γ is FDA-approved for the treatment Chronic Granulomatous Disease patients at risk of invasive fungal and other infections in combination with antifungal therapy and Granulocyte-macrophage colony-stimulating factor (GM-CSF) (347).

#### Colony Stimulating Factors

In cancer patients with chemotherapy-associated neutropenia, the prophylactic use Granulocyte Colony-Stimulating Factor (G-CSF; e.g., filgrastim) is FDA-approved and results in a decrease in rates of infection and infection-related morbidity (all causes) in patients receiving cancer therapy or undergoing stem-cell transplantation, although the effect on infection-related mortality was moderate (348). In a clinical study of patients with haematological malignancy and suspected or proven systemic fungal infection, nearly twice as many responded to amphotericin B therapy with concomitant G-CSF compared to those receiving amphotericin B alone (349). Another small study (8 patients with leukaemia (*n* = 7) or breast cancer (*n* = 1) demonstrated that adjuvant therapy with G-CSF in addition to amphotericin B resulted in cure (*n* = 4), partial response (*n* = 2) or failure (*n* = 2), indicating potential utility of G-CSF in resolving fungal infection in patients with malignancy (350). In another study, G-CSF in combination with fluconazole resulted in faster infection resolution in non-neutropenic patients with invasive candidiasis/candidemia (324, 351). Treatment with G-CSF before chemotherapy resulted in a dose-dependent increase in the number of neutrophils and treatment after chemotherapy initiation reduced the number of days on which the neutrophil count was ≤1,000/μl, the number of days on which antibiotics were used to treat fever and the incidence and severity of mucositis was decreased (352). G-CSF also enhanced the respiratory burst response of human phagocytes *in vitro* to fungal conidia or yeast cells, but not hyphae (353).

Granulocyte-macrophage colony-stimulating factor (GM-CSF; e.g., sargramostim) promotes neutrophil, monocyte, macrophage and lymphocyte production, maturation, activation and migration (as well as progenitor cells), whereas G-CSF primarily affects myeloblasts and neutrophils and M-CSF primarily affects only monocytes. GM-CSF is licensed for the treatment of chemotherapy-associated neutropenia and stem cell transplantation (354, 355) and is likely to have advantages over G-CSF therapy due to its wider effects on fungi and the immune system (324). In a randomized trial of patients receiving allogenic haematopoietic stem cell transplantation HSCT, 100-day cumulative mortality and 100-day transplantation-related mortality were lower in patients receiving GM-CSF than receiving G-CSF and after 600 days of follow-up infection-related mortality and invasive fungal disease-related mortality was lower in the GM-CSF group compared to the G-CSF group (355). In other studies of acute myeloid leukaemia patients, administration of GM-CSF led to recovery of neutrophil counts and was associated with a more rapid clearance of infection when compared with a historical control group that did not receive GM-CSF (356), including fungal infections (357). In a small study of neutropenic patients with fungal infection, eight patients received amphotericin B and GM-CSF. Six patients responded to treatment, with four undergoing complete recovery, whereas the remaining two patients died of fungal infection. Although this study did not have controls, the survival rate is higher than would be infected from antifungal treatment alone (358). In a study of 11 AIDS patients with fluconazole-refractory oropharyngeal candidiasis that received GM-CSF and fluconazole, a mycological response was seen in seven patients and three patients were cured (359). Three patients with rhinocerebral zygomycosis were successfully treated with adjunctive GM-CSF when added to antifungal therapy (amphotericin B) and surgery (360). However, in a study of acute myelogenous leukaemia in elderly patients (55–75 years), GM-CSF therapy (114 patients) did not improve complete remission rates when compared to patients receiving placebo (126 patients), but did prolong disease-free survival and overall survival. The number of patients with infections, including serious fungal infections, was not different between the GM-CSF and placebo groups (361).

Macrophage Colony-Stimulating Factor (M-CSF) can rapidly increase myeloid differentiation of haematopoietic stem cells. In a study of bone marrow transplant patients that developed invasive fungal infection and that received recombinant human M-CSF (rhM-CSF), survival was greater than historical patients not receiving rhM-CSF with *Candida* spp. infection, but not in patients with *Aspergillus* spp. infection or in any patients with Karnofsky scores of <20% (362, 363). Exogenous M-CSF was protective in murine models of *Aspergillus* spp. and *Pseudomonas aeruginosa* infection following haematopoietic stem cell or progenitor cell transplantation and was more efficacious than G-CSF (364). Synergy of M-CSF with fluconazole was observed in human monocyte-derived macrophages infected with *Cryptococcus neoformans*. M-CSF alone also reduced counts of *Cryptococcus neoformans* in this model (365) and in a murine model of *Cryptococcus neoformans* infection (366). In a rat model of acute candidiasis, administration of ≥0.1 mg/kg M-CSF with 0.3 mg/kg fluconazole enhanced survival (>30 d) compared with fluconazole alone (5 d) and similarly reduced *C. albicans* kidney burden in a chronic model of candidiasis (367). Conversely, another study of mice infected intravenously with *C. albicans* demonstrated that treatment with M-CSF exacerbated disease and led to significantly earlier death (368). Clearly, M-CSF has potential in the treatment of invasive fungal infection, either alone, or in combination with antifungal therapy, but more research is clearly required and it is possible that the effect may be dependent on the infecting pathogen. Thus, whilst showing clear promise the use of colony-stimulating factor therapy should be the subject of appropriately controlled clinical studies in patients with accurately diagnosed fungal infections and comparable antifungal therapeutic regimens.

### Immune Checkpoint Inhibitors

Immune checkpoints are important regulators of immune homeostasis. Immune checkpoints consist of both stimulatory and inhibitory pathways that are important for maintaining self-tolerance and regulating the type, magnitude, and duration of the immune response (369, 370). Immune checkpoint therapies in oncology target regulatory pathways in T cells to enhance anti-tumour responses (370–373) and are used for the treatment of squamous-cell carcinoma and advanced melanoma. The checkpoint programmed cell death 1 (PD1) (a member of the B7-CD28 superfamily) is expressed on monocytes, natural killer cells, T- and B-lymphocytes. Binding of PD1 to the ligand PD1-L1 on myeloid cells impairs T-cell functions including cytokine production and cytotoxic activity, whereas blocking binding of PD1 to its ligand with an anti-PD1 antibody can restore immune function. Cytotoxic T lymphocyte-associated antigen-4 (CTLA-4) is another immune checkpoint that can impair T-cell function and Ipilimumab (an anti-CTLA-4 antibody) was the first immune checkpoint inhibitor approved for the treatment of cancer (374). The PD1 and CTLA-4 pathways have roles to play in antifungal defences (374), as demonstrated *in vitro* in a murine model of *Histoplasma capsulatum* infection (PD1) (375) and in blood from patients with paracoccidioidomycosis (CTLA-4) (376). In a murine model of *C. albicans* sepsis, antibodies to PD1 and PD-L1 were effective at improving survival, as was an antibody to CTLA-4 in this model (377) and in a murine model of *Cryptococcus neoformans* infection (378). Nivolumab, an antibody drug that blocks PD1, was used successfully in combination with IFN-γ and antifungal therapy (liposomal amphotericin B and posaconazole) in a case of invasive mucormycosis following unsuccessful antifungal therapy for 28 days (379). The use of Nivolumab for immune checkpoint inhibition in sepsis (documented or suspected infection) has been the subject of a recent Phase 1b clinical trial (NCT02960854) (380).

### Vaccines

It is estimated that vaccination prevented at least 10 million deaths globally between 2010 and 2015 (381). No fungal vaccine has yet been approved for use in humans although clinical trials of fungal vaccines have been reported and a number are in preclinical and clinical development (382, 383). Our ever-improving knowledge of the immune system ought to increase the likelihood of developing fungal vaccines, but a number of challenges exist and for a number of infectious diseases, treatment rather than vaccination remains the optimal strategy. Eliciting a protective response to immunisation in immunocompromised individuals who have developed/are at risk of invasive fungal infection is unlikely, particularly without risk of aggravating underlying disease and/or development of the fungal infection due to attenuated vaccine administration (382–384). Additionally, developing a vaccine against commensal microorganisms, e.g., *Candida* spp. could represent an additional problem (385). The high costs associated with vaccine development are a challenge considering that revenue will only be obtained from vaccinating only populations at risk of developing fungal infection, or in the case of endemic mycosis, only a limited patient population cannot attract sufficient investment (386).

A vaccine (NDV-3A) containing the N-terminal portion of the agglutinin-like sequence 3 (Als3) protein of *C. albicans*, is in development by NovaDigm Therapeutics for the prevention of recurrent vulvovaginal candidiasis (VVC). Als3 is a hyphal-specific virulence factor that mediates adherence to and invasion of human epithelial and vascular endothelial cells. In a Phase II randomized, double-blind, placebo-controlled clinical trial, NDV-3A demonstrated a statistically significant increase in the percentage of symptom-free patients at 12 months after vaccination and a doubling in the median time to first symptomatic episode for a subset of patients aged <40 years (ClinicalTrials.gov Identifiers: NCT01926028 and NCT02996448) (382, 387). Another vaccine, PEV7, has been the subject of a successful Phase I clinical trial (ClinicalTrials.gov Identifier: NCT01067131) for the prevention of recurrent VVC. PEV7 was developed by Pevion Biotech (rights subsequently acquired by NovaDigm Therapeutics) and contains recombinant secreted aspartyl protease 2 (rSAP-2) incorporated into influenza virisomes. Trial results demonstrated the generation of specific and functional B cell memory in 100% of the vaccinated women and a favourable safety profile (388). Earlier reports of an oral vaccine, D.651, for the prevention of VVC recurrence was prepared using ribosomes of *C. albicans* serotypes a and b. A Phase II clinical trial reported a good safety profile and efficacy, in which 13 of 20 patients taking the vaccine did not experience recurrence of VVC during the 6 months taking the vaccine (389). The current status of this vaccine is not known. In another study, a vaccine consisting of formaldehyde-killed spherules of *Coccidioides immitis* was tested in humans, but a statistically significant reduction of the incidence of infection was not observed in those vaccinated (390). A number of other fungal vaccines have been tested in animal models and are beyond the scope of this manuscript, but have been the subject of several recent reviews (328, 383, 388, 391–394). The vaccines described above represent the only ones to reach clinical trials to the best of the authors knowledge.

On a cautionary note, in some cases, live, attenuated fungi (*Blastomyces dermatitidis* and *Histoplasma capsulatum*) have demonstrated the induction of protective immunity in mice (395). Naturally, caution would be required before testing live attenuated fungi in immunocompromised individuals although live, attenuated vaccines are arguably much more appropriate candidates for vaccination against endemic fungal infections, such as histoplasmosis and sporotrichosis, in otherwise immunocompetent, healthy subjects.

Interestingly, heat-killed *Saccharomyces cerevisiae* administered as a vaccine was protective against systemic aspergillosis, candidiasis, cryptococcosis and coccidioidomycosis in mouse models (396), but to the best of our knowledge has not yet been tested in humans.

### Antifungal Monoclonal Antibodies

Monoclonal antibodies (mAb) represent some of the world's best-selling therapeutics, of which more than 80 have received marketing approval and more than 100 are in development. In 2018 alone, twelve new mAb were approved by the FDA, representing 20% of the total number of approved drugs and sales of mAb were forecast to reach US $125 Bn by 2020. Most therapeutic monoclonal antibodies are used for the treatment of cancer or immunological disorders (397, 398). The development of monoclonal antibodies for the prevention and treatment of infectious diseases lags somewhat behind their development for other therapeutic areas, e.g., cancer and autoimmune diseases (399), and only three monoclonal antibodies have received approval for infectious disease prophylaxis or treatment; palivizumab for prevention of respiratory syncytial virus in high-risk infants (400); and obiltoxaximab (401) and raxibacumab (402) for prophylaxis and treatment of anthrax. The lack of development of mAb for infectious diseases may be because consensus on clinical end-points and definitions on conditions of use are lacking, as well as high costs associated with their development and lack of a cle arly defined market for these products (399). Fungal-specific mAb can mediate protection from fungal infection by direct action on fungal cells or via promotion of phagocytosis and complement activation. However, some mAb to fungi can be disease-enhancing or have no effect (403). Protective mAb against human fungal pathogens are currently in preclinical development (382), including examples with narrow spectrum reactivity [e.g., mAb 3D9.3 (anti-Als3) that specifically recognises *C. albicans* (404)] and broad-spectrum reactivity with a number of fungal pathogens (e.g., mAb C7 (anti-Als3) which inactivates germ tubes and spores of *Candida* spp., *Cryptococcus neoformans, A. fumigatus* and *Scedosporium prolificans* (405).

A murine mAb, 18B7, was raised against *Cryptococcus neoformans* and bound to capsular glucuronoxylomannan in infected mouse tissues (406). 18B7 was protective in a murine intraperitoneal model of *Cryptococcus neoformans* infection (407). In a human Phase I dose escalation study of human immunodeficiency virus (HIV)-infected patients who had been successfully treated for cryptococcal meningitis, the maximum tolerated dose was established as 1.0 mg/kg and serum cryptococcal antigen titres declined by a median of 3-fold at 2 weeks post-infusion. However, titres subsequently returned toward the baseline values by week 12, 3 of 4 subjects in the 1.0-mg/kg dosing cohort had a 0.5 log10 increase in HIV load and 18B7 was not detected in cerebrospinal fluid (408).

Interestingly, Rudkin et al. generated the first set of fully human anti-*Candida* spp. mAb isolated from B cells of patients suffering from candidiasis and that demonstrated morphology-specific, high avidity binding to the cell wall, including mAb specific for the *C. albicans* hyphal cell wall protein Hyr1. Cell wall mAb demonstrated cross-reactivity with other *Candida* spp., whereas anti-Hyr1 mAb were cross-reactive with only *C. albicans*. Importantly, tested mAb promoted phagocytosis of *C. albicans* by macrophages and reduced fungal burden in therapeutic or prophylactic murine models of disseminated candidiasis (409), but these have yet to be tested in humans. Efungumab (Mycograb) is a recombinant human mAb against fungal HSP90 with activity against *C. albicans, C. krusei, C. tropicalis, C. glabrata*, and *C. parapsilosis* (410, 411). *In vitro* studies revealed synergy with fluconazole, amphotericin B (AmB) and caspofungin, and in a murine model of systemic candidiasis, efungumab improved the killing of *Candida* spp. (*C. albicans, C. krusei*, and *C. glabrata*) in combination with AmB (412). However, the combination effect of efungumab and AmB was later revealed to be a nonspecific protein effect, as addition of efungumab or other unrelated proteins, including human serum, resulted in similar decreases in the MIC of AmB (413). Although clinical trials of this product were conducted, they were unsuccessful and development of this drug candidate has been abandoned.

Therefore, the potential for the use of mAb for treatment or prophylaxis against fungal infection remains a possibility, but large-scale clinical trials will be required to bring this promise to fruition.

## Conclusions

Antimicrobial peptides are promising candidates as therapeutics for the treatment of fungal infection and are much needed in clinical practice due to the limited array of treatment options and increasing resistance to existing antifungals. Unfortunately, we are not seeing enough drug candidates making it through the drug development pipeline, as *in vitro* and *in vivo* testing approaches are not always appropriate and/or optimised for AMP (268). The same is true in part for clinical efficacy trials which must be appropriate for AMP (end points in particular). These factors are undoubtedly part of the reason behind there not being more AMP progressing through the drug development cycle and/or AMP candidates are confined to topical therapy status as delivery systems, formulation, routes of administration and duration of therapy for AMP have not been adequately optimised. The time is now coming for greater exploitation of AMP and other immunotherapeutics as antifungal drug candidates as we gain a greater understanding of how best to test these drug candidates *in vitro* and how regulatory pathways and clinical studies can be more accommodating for peptides (Table 3). As the global AMR crisis worsens and emerging fungal diseases increase, the potential of these drug candidates must be fulfilled sooner rather than later.

**Table 3:** Selected immunology-based approaches for the treatment of fungal infection.

**Antifungal therapy**	**Target fungal infection**	**Developmental therapeutic**	**Target pathogen/s**	**Development stage as antifungal**	**Reference/ ClinicalTrials.gov identifier**
**AMP**					
Antifungal	Onychomycosis	NP213	Dermatophytes	Phase IIb	(173)
	Onychomycosis	HXP124	Dermatophytes	Phase I/IIa	(174)
	VVC^1^	CZEN-002	*Candida* spp.	Phase I/IIa	(56)
	Oral candidiasis	P113	*Candida* spp.	Phase IIb	NCT00659971
	Dermal infection	Omiganan	*Candida* spp.	*In vivo* (porcine)	(209)
	Prophylaxis in HSCT^2^ patients	hLF1-11	Not Specified	Phase I	(225)
	Oral mucositis	Iseganan	Yeasts	Phase III	(240)
	Not specified	LTX-109	*S. cerevisiae*	*In vitro*	(245)
	Aspergillosis & Candidiasis	NP339	*Aspergillus* spp., *Candida* spp., mucorales	*In vitro*	(290)
	Fungal infection	D2A21	*Mucor* spp., *T. mentagrophytes*	*In vitro*	(259)
	Systemin infection	ETD151	*C. albicans, A. fumigatus*	*In vivo* (murine)	(263)
Anti-biofilm	Not specified	Histatin-5	*C. albicans*	*In vitro*	(83)
	Not specified	LL-37	*C. albicans*	*In vitro*	(128)
	Not specified	hLF1-11	*C. albicans*	*In vitro*	(215)
	Not specified	LTX-109	*S. cerevisiae*	*In vitro*	(245)
**Immunostimulatory molecules**				Clinical	
Interferon-γ	Systemic infection	IFN- γ	*Aspergillus* spp., *Candida* spp.		(347)
Colony stimulating factors	Prophylaxis	G-CSF^3^	Fungal	Clinical	(348)
	Prophylaxis	GM-CSF^4^	Fungal	Clinical	(355)
	Prophylaxis	M-CSF^5^	*Candida* spp.	Phase I/II	(363)
**Antibodies**
Prophylaxis	Cryptococcosis	18B7	*Cryptococcus neoformans*	Phase I	(408)
	Candidiasis	mAb 3D9.3	*C. albicans*	*In vitro*	(404)
	Fungal	mAb C7	*Candida* spp., *Cryptococcus* spp., *A. fumigatus, Scedosporium prolificans*	*In vitro*	(405)
Therapeutic	Disseminated candidiasis	Ab119 & Ab120	*Candida* spp.	*In vivo* (murine)	(409)
**Vaccines**	VVC^1^	NDV-3A	*Candida* spp.	Phase II	(387)
	VVC^1^	PEV7	*Candida* spp.	Phase I	(388)
	VVC^1^	D.651	*Candida* spp.	Phase II	(389)
**Immune checkpoint inhibitors**	Mucormycosis	Nivolumab	Mucorales	Case study (1 patient)	(379)
**Cell-based therapies**					
Antifungal-loaded leukocytes	Pulmonary aspergillosis	Posaconazole-loaded leukocytes	*Aspergillus* spp.	*In vivo* (murine)	(414)
CAR-T	Murine lung infection	D-CAR^+^ T cells^6^	*Aspergillus* spp.	*In vivo* (murine)	(415)

1 *Vulvovaginal candidiasis*.

2 *Haematopoietic stem cell transplantation*.

3 *Granulocyte Colony-Stimulating Factor*.

4 *Granulocyte-Macrophage Colony-Stimulating Factor*.

5 *Macrophage Colony-Stimulating Factor*.

6 *Dectin-Chimeric Antigen Receptor Positive T-cells*.

## Author Contributions

DM and DO'N contributed to the writing and editing of this manuscript. All authors contributed to the article and approved the submitted version.

## Conflict of Interest

DM is an employee of NovaBiotics Ltd., and holds stock options. DO'N is a Director, shareholder, and employee of NovaBiotics.

## Footnotes

^1^Antifungal Drugs Market by Drug Class (Azoles, Echinocandins, Polyenes, Allyamines, Others), Indication, Dosage Form, Regions, Global Industry Analysis, Market Size, Share, Growth, Trends, and Forecast 2019 to 2026. Available online at: https://www.fiormarkets.com/report/antifungal-drugs-market-by-drug-class-azoles-echinocandins-407129.html
